# Differential regulation of STING expression and cisplatin sensitivity by autophagy in non-small cell lung cancer cells

**DOI:** 10.1007/s12032-025-02786-2

**Published:** 2025-05-30

**Authors:** Sevim Aydemir, Zafer Yildirim, Busra Bara, Eda Dogan, Vildan Bozok

**Affiliations:** Faculty of Medicine, Department of Medical Biology, Ege University, Izmir, Türkiye

**Keywords:** cGAS-STING, Autophagy, Chloroquine, Starvation, Cisplatin, NSCLC

## Abstract

The cGAS-STING pathway is a central signalling mechanism in inflammatory responses and can be activated by cisplatin. Increased autophagic activity has been linked to cisplatin resistance in non-small cell lung cancer (NSCLC); however, how autophagy-STING interactions influence the cisplatin response remains unclear. This study investigates how autophagy modulation affects STING expression and cisplatin sensitivity in NSCLC cells with different basal STING levels. Autophagy was inhibited using chloroquine and induced by serum starvation in Calu-1 and H2030 cells. In Calu-1 cells, cisplatin treatment increased STING expression, activated the cGAS-STING pathway, and induced interferon responses correlated with cisplatin concentration. Autophagy inhibition reduced STING expression and interferon activation while enhancing cisplatin sensitivity. Conversely, autophagy induction caused fluctuations in STING expression and decreased cisplatin sensitivity, with ISG15 expression being selectively increased under serum starvation. In contrast, H2030 cells exhibited low basal STING expression and showed minimal responses to cisplatin or autophagy modulation. These findings suggest that STING expression levels critically influence autophagy-mediated responses to DNA-damaging chemotherapy in NSCLC.

**Supplementary Information:**

The online version contains supplementary material available at 10.1007/s12032-025-02786-2.

## Introduction

The cyclic GMP-AMP synthase (cGAS)-stimulator of interferon genes (STING) signalling pathway is the primary mediator of the DNA-triggered immune response in the cytoplasm of mammalian cells [1]. cGAS binds to endogenous or exogenous DNA, upon undergoing a conformational change to reach its active state, catalyses adenosine triphosphate (ATP) and guanosine triphosphate (GTP) to produce cyclic guanosine adenosine monophosphate (cGAMP), a second messenger that binds and activates STING on the endoplasmic reticulum (ER) membrane. Activated STING is then transferred to the Golgi, where it activates tank binding kinase-1 (TBK1) and IFN regulatory factor-3 (IRF3). Upon phosphorylation, IRF3 forms a dimer and translocate into the nucleus to trigger the transcription of IFN-I and pro-inflammatory cytokines.

Autophagy serves as a crucial self-defence mechanism, responsible for the removal of misfolded or aggregated proteins, the clearance of damaged organelles, and the elimination of intracellular pathogens [2]. It is controlled by nearly 40 Autophagy Related Genes (ATGs), which were first identified in yeast and have mammalian homologs [3]. The conversation of LC3B-I, which is the mammalian homolog of ATG8, to LC3B-II serves as a marker for the presence of autophagy [4]. It has been reported that STING localised in the ER-Golgi intermediate compartment (ERGIC) provides a membrane source for LC3 lipidation [5].

Cisplatin, a platinum-based chemotherapy agent, is the first line therapy used in the treatment of various cancers, including non-small cell lung cancer (NSCLC) [6]. It binds to purine bases in DNA, forming DNA breaks or adduct structures, and induces apoptosis [7]. Previous studies have shown that, autophagy is induced during the development of cisplatin resistance in Calu-1 cells [8]. Inhibition of autophagy has been found to enhance the cisplatin response [9–11] and overcame cisplatin resistance [12]. However, autophagy is defined as Janus-faced in terms of carcinogenesis, as it can have cyto-protective, cytotoxic, cytostatic and non-protective functions [13].

cGAS-STING pathway-activated NSCLCs exhibited a hot immune microenvironment including higher levels of targetable immune checkpoints associated with clinical response to immunotherapy [14]. Furthermore, cisplatin treatment has been shown to accelerate cGAS-STING activation in NSCLC [14]. Hybrid platinum prodrug loaded nanoparticles have been found to cause DNA double damage and activate the cGAS-STING pathway, promote dendritic cell maturation and increase cytotoxic T lymphocyte infiltration in colorectal cancer [15]. cGAS-STING mediated immunogenicity triggered by DNA damage has also been reported in breast and small cell lung cancer (SCLC) [16, 17]. Furthermore, dysregulated autophagic flux has been implicated in promoting drug resistance in various cancers, including during chemotherapy [18]. However, it remains unclear whether autophagy and cGAS-STING activation play a role in cisplatin response. Therefore, this study aimed to investigate how modulation of autophagy affects STING expression, interferon responses, and cisplatin sensitivity in NSCLC cells with different basal STING levels.

## Material methods

### Cell and chemicals

Calu-1 and H2030 NSCLC cell lines were obtained from ATCC and cultured using Dulbecco’s Modified Eagle’s Medium (DMEM) and RPMI-1640 medium, respectively. Media were supplemented with 10% Foetal Bovine Serum (FBS), 1% penicillin/streptomycin, and 1% L-glutamine. Cisplatin was 50 mg/50 mL stock concentration (Cipintu). Chloroquine diphosphate salt (#C6628, Sigma-Aldrich) was dissolved in distilled water at 30 mM. Monodansylcadavarine (MDC) was obtained from Sigma-Aldrich (#D4008).

### Cytotoxicity assay

The XTT cell proliferation kit (#203001000, Biological Industries) was used to determine the half-maximal inhibitory concentration (IC_50_) of cisplatin in Calu-1 and H2030 cell lines. Briefly, 4 × 10^3^ cells were prepared in 96-well plates and cisplatin concentrations of 60-50-40-30-20-10-5 μM was applied after cell attachment. After 24, 48, 72 h of exposure, cell viability was measured. The mean absorbance for each concentration was normalized to the control, and % viability was calculated using GraphPad Prism 9.3.1. software.

### Modulation of autophagy

Chloroquine (CQ) was used to inhibit of autophagy in Calu-1 and H2030 cells. To examine the CQ’s inhibitory effects, 3 × 10^5^ cells/well were seeded in a 6-well plate. After cells adherence, 50 μM CQ was prepared in medium containing 10% FBS under dark conditions and added to the wells in a total volume of 2 ml. After incubation with CQ for 48 h, RNA and protein were isolated from both control and treatment groups. Serum starvation method was used to induce autophagy [19]. For this purpose, cells were seeded in 6-well plates at 3 × 10^5^ per well. After 24 h, the cells were maintained in fresh medium without serum and amino acids for 4, 6, 8, and 24 h.

### Detection of autophagosomes

MDC autofluorescent dye was used to stain autophagosomes [20]. For this, 3 × 10^5^ cells/well were seeded in a 6-well plate. After 24 h of incubation, 50 µM MDC was added and incubated for 1 h at 37 °C. Cells were then fixed using 4% paraformaldehyde in PBS for 20 min and visualized using fluorescence microscopy. Imaging was performed on an Olympus BX41 fluorescence microscope equipped with a 360 nm excitation filter and a 525 nm emission filter. Images were captured using a CCD camera (Olympus DP70). The proportion of MDC-positive cells was assessed by analysing 100 cells per group, independently scored by two researchers [21].

### Western blot

Proteins were isolated using cOmplete™ Lysis-M (#4719956001, Roche). 20 µg protein samples were separated via Sodium Dodecyl Sulphate (SDS) polyacrylamide gel electrophoresis and transferred to a PVDF membrane. The following primary antibodies were used at 1:1000 dilution: ATG13 (#13468, Cell Signaling), β-actin (#sc47778, Santa Cruz), IRF3 (#66670, Cell Signaling), LC3A (#4599, Cell Signaling), LC3B (#18725, Proteintech), STING (#66680, Proteintech), p-STING (#19781, Cell Signaling). HRP-conjugated Goat Anti-Mouse IgG (#SA00001, Proteintech) and Anti-rabbit IgG, HRP-linked Antibody (#7074S, Cell Signaling) were used as seconder antibodies.

### qRT-PCR

mRNA levels of target genes were analysed using qRT-PCR. Aurum™ Total RNA Mini Kit (#7326820, Bio-Rad) and iScript™ cDNA Synthesis Kit (#1708840, BioRad) were used for total RNA isolation and cDNA synthesis. iTaq Universal SYBR Green Supermix (#1725120, Bio-Rad) protocol and LightCycler480 instrument were used for qRT-PCR analysis. Data were normalized to untreated (UT) control, using 18S rRNA as the reference housekeeping gene. The results were analysed using the comparative 2^−ΔΔCt^ method. Primer sequences are provided in Table 1.

**Table 1:** Sequences of primers used at qRT-PCR (5′…3′)

18S rRNA	F: GATGGGCGGCGGAAAATAG
	R: GCGTGGATTCTGCATAATGGT
STING	F: GCAGTGTGTGAAAAAGGGAAT
	R: CACCCCGTAGCAGGTTGTT
IRF3	F: AGAGGCTCGTGATGTGGTCAAG
	R: AGGTCCACAGTATTCTCCAGG
IFI44	F: GATGTGAGCCTGTGAGGTCC
	R: CTTTACAGGGTCCAGCTCCC
IFIT2	F: GCGTGAAGAAGGTGAAGAGG
	R: GCAGGTAGGCATTGTTTGGT
IL6	F: AGACAGCCACTCACCTCTTCAG
	R: TTCTGCCAGTGCCTCTTTGCTG
ISG15	F: CAGCCATGGGCTGGGAC
	R: GCCGATCTTCTGGGTGATCT

### Statistical analyses

Statistical analyses were performed using GraphPad Prism software (version 9.3.1). Student’s t-test and one-way ANOVA were used where appropriate. For multiple comparisons against the control group, Dunnett’s post-hoc test was applied. IC_50_ values for cisplatin were determined from dose–response curves using nonlinear regression analysis. A p-value of less than 0.05 was considered statistically significant.

## Results

### Cisplatin activated STING pathway in Calu-1 cells

**Fig. 1:**
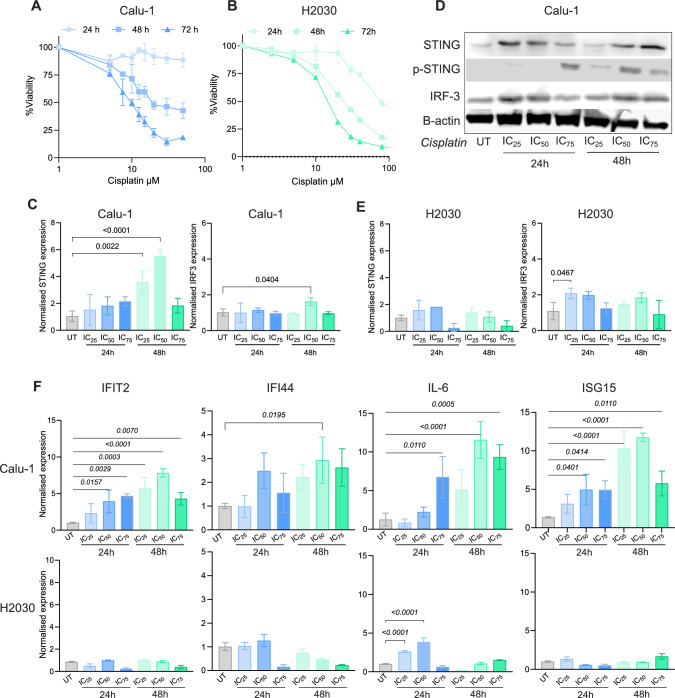
Cisplatin treatment induces STING expression in epidermoid carcinoma but not adenocarcinoma NSCLC cells. Cytotoxic effects of cisplatin in **A** Calu-1 and **B** H2030 cell lines; **C** mRNA expression results of STING and IRF3 in Calu-1 cells; **D** Western blot results for STING, p-STING and IRF3 proteins in Calu-1 cells. Β-actin was used as loading control; **E** mRNA expression results of STING and IRF3 in H2030 cells; **F** qRT-PCR results for IFIT2, IFI44, IL6, and ISG15 genes in Calu-1 and H2030 cells. The IC_25_, IC_50_ and IC_75_ treatment doses are; 4.58 µM, 9.16 µM and 13.75 µM for Calu-1 cells and 6.76 µM, 13.52 µM and 20.28 for H2030 cells, respectively. p-values of significant comparisons were indicated on the graphs using ANOVA and Dunnett’s multiple comparisons test

Cisplatin exhibited dose- and time-dependent cytotoxic effects on both Calu-1 and H2030 cell lines, with IC_50_ values at 72 h were determined as 9.16 µM and 13.52 µM, respectively (Fig. 1A, B). Cells were treated with cisplatin at IC_25_, IC_50_, and IC_75_ concentrations, and the expression of cGAS-STING pathway components and interferon stimulated genes was analysed.

In Calu-1 cells, STING mRNA expression significantly increased in a cisplatin concentration-dependent manner, peaking at the 48 h IC_50_ dose (p < 0.0001). However, started to decline at the 48 h IC_75_ dose (Fig. 1C). Western blot analyses confirmed these findings, showing altered STING expression in response to cisplatin dose and exposure time as shown in Fig. 1D and Suppl. Figure 1A. Additionally, phosphorylated STING protein was detected at the 24 h IC_75_ and 48 h IC_25-50-75_ concentrations confirming the activation of the cGAS-STING pathway (Fig. 1D, Suppl. Figure 1B). A slight increase in IRF3 mRNA and protein expressions were detected in Calu-1 cells (Fig. 1C, D, Suppl. Figure 1C).

In contrast, qRT-PCR results showed that H2030 cells exhibited low STING mRNA levels, and no STING protein was detected via western blot analysis (Fig. 1E and Suppl. Figure 1D). A modest increase in IRF3 mRNA expression was observed at 24 h IC_25_ group (p = 0.0467) (Fig. 1E).

To assess the interferon response following cisplatin treatment, we analysed IFIT2, IFI44, IL-6, and ISG15 expression. In Calu-1 cells, all four genes were upregulated (Fig. 1F). However, H2030 cells exhibited a limited interferon response, with only IL-6 expression significantly increasing at 24 h IC_25_ and IC_50_ (p < 0.0001) (Fig. 1F).

These results confirm that cisplatin treatment induces STING expression and activates the interferon response in Calu-1 epidermoid carcinoma cells. In contrast, H2030 adenocarcinoma cells lack STING protein, and cisplatin does not induce STING expression or a strong interferon response. Given these differences, Calu-1 and H2030 cells serve as suitable models to investigate how autophagy modulation affects cisplatin sensitivity and the interferon response in a STING-dependent manner.

### Cisplatin treatment induced autophagy in Calu-1 cells

**Fig. 2:**
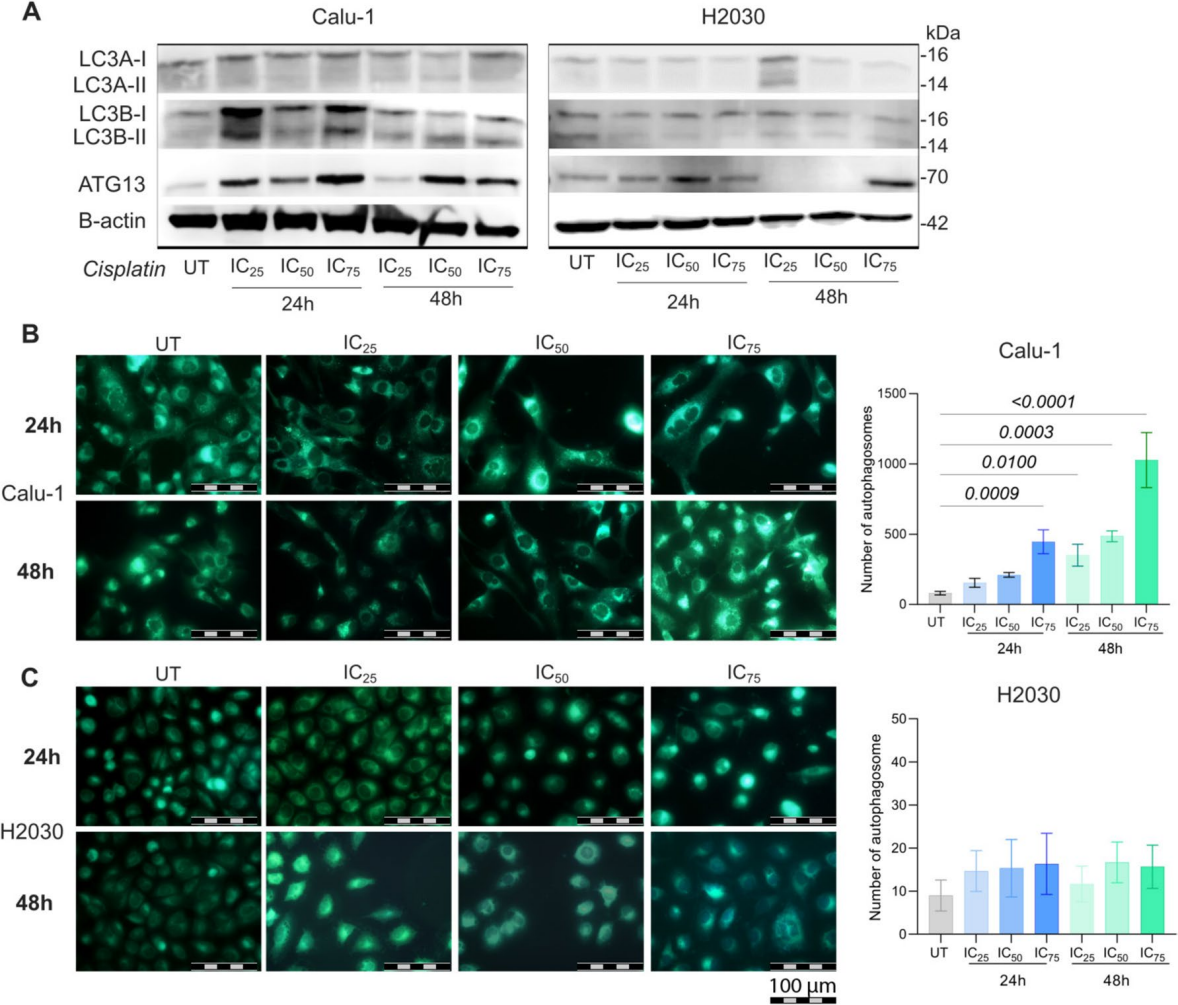
Cisplatin treatment induces autophagy in Calu-1 epidermoid carcinoma cells. Cisplatin was applied to the cells at IC_25_, IC_50,_ and IC_75_ concentrations for 24–48 h. **A** Western Blot results for LC3A, LC3B, ATG13, and B-actin proteins in Calu-1 and H2030 cells. **B** MDC staining of autophagosomes and bar graph of the autophagosome ratios in **B** Calu-1 and **C** H2030 cell line (n = 100). Scale bar = 100 µm. The same β-actin loading control is shown in Figs. 1(D) and 2(A) as the membrane was stripped and re-probed for multiple target proteins

To evaluate the effect of cisplatin treatment on autophagy, LC3A, LC3B and ATG13 proteins were analysed by western blot and autophagosome formation was assessed using MDC staining after treating cells with IC_25_, IC_50_, and IC_75_ cisplatin concentrations for 24 and 48 h. In Calu-1 cells, LC3A expression remained unchanged between control and cisplatin-treated groups (Fig. 2A). However, LC3B-II conversion and ATG13 expression increased, particularly at 24 h post-treatment (Fig. 2A), indicating autophagy induction.

In contrast, H2030 cells showed a decrease in LC3B-II conversion following cisplatin treatment compared to untreated controls, with very low LC3B-II levels detected in cisplatin-treated cells (Fig. 2B). ATG13 expression was not detected at the IC_25_ and IC_50_ doses of 48 h treatment in H2030 cells.

MDC selectively accumulates in the autophagic vacuoles, allowing for visualization of autophagosome formation [20]. In Calu-1 cells, autophagosomes significantly increased with cisplatin concentration and exposure time. The highest increase was seen at the 48 h IC_75_ dose, where a 946.7 ± 72.04-fold increase was observed compared to the control (p < 0.0001) (Fig. 2C). In contrast, H2030 cells exhibited no significant changes in autophagosome formation (Fig. 2D). These findings confirm that cisplatin induces autophagy in Calu-1 cells but not in H2030 cells, suggesting a potential link between STING expression and autophagy induction in response to cisplatin treatment.

### Impact of autophagy inhibition on cisplatin sensitivity, STING expression, and interferon response

**Fig. 3:**
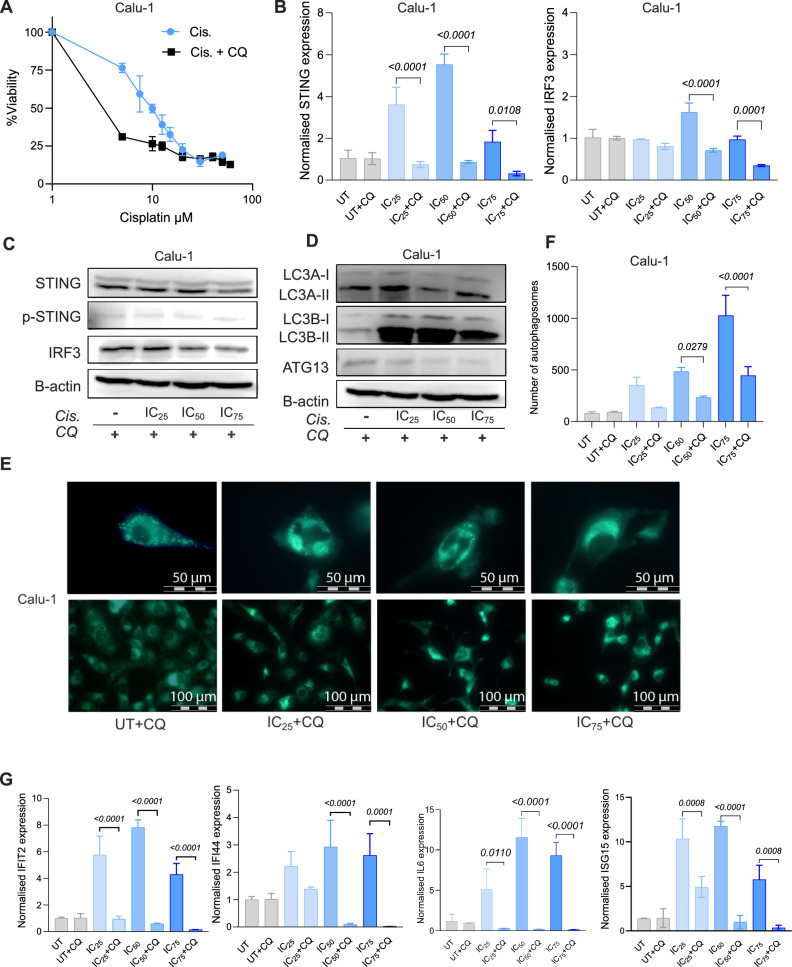
Impact of inhibition of autophagy on cisplatin sensitivity, STING expression and interferon response. **A** Viability curves of cisplatin (Cis.) and Cis. + Chloroquine (CQ) treated Calu-1 cells. Cisplatin doses: 60-50-40-30-20-10-5 μM and CQ dose: 50 µM; **B** qRT-PCR results of STING and IRF3 gene expression in Calu-1 cells. Cisplatin concentrations: 4.58, 9.16, 13.75 µM for IC_25_, IC_50_ and IC_75_ groups, respectively. CQ dose: 50 μM. **C** Protein levels of STING, p-STING and IRF3 in Calu-1 cells. β-actin protein used as a loading control. **D** Protein levels of autophagy markers (LC3A-II, LC3B-II, and ATG13) analysed by Western Blot. 20 μg total protein was loaded for all Western Blot analysis. **E** MDC staining of autophagosomes, Scale bar = 50 µm and 100 µm. **F** Bar graph showing the number of autophagosomes (n = 100 cells). **G** mRNA expression levels of IFIT2, IFI44, IL6, and ISG15 genes. Significant p values were calculated using ANOVA and Dunnett’s multiple comparisons test

To assess the effects of autophagy inhibition on cisplatin sensitivity, STING expression and interferon response, Calu-1 cells were treated with cisplatin combined with chloroquine (CQ). Our results showed that inhibition of autophagy enhanced cisplatin sensitivity (Fig. 3A). Specifically, the IC_50_ value for cisplatin alone in Calu-1 cells was 9.16 µM, which decreased to 4.36 µM with the addition of CQ, indicating an increased sensitivity to cisplatin.

Although cisplatin treatment significantly increased STING mRNA levels (Fig. 1C), autophagy inhibition with CQ resulted in a substantial reduction in STING expression in all Cis. + CQ treatment groups (Fig. 3B). Similarly, IRF3 mRNA expression was significantly decreased in the IC_50_ + CQ and IC_75_ + CQ treatment groups (Fig. 3B). Compared to the CQ− control, where only autophagy was inhibited, the total STING and IRF3 protein levels were notably reduced in IC_75_ + CQ group (Fig. 3C, Suppl. Figure 2A, B). However, slight p-STING expression was similar between control and treated cells (Fig. 3C).

LC3A-II accumulation was observed upon CQ treatment, with an approximately 1.6-fold increase detected in the IC_25_ + CQ group (Fig. 3D, Suppl. Figure 2C.). Inhibition of autophagy with CQ also resulted in LC3B-II accumulation: however, the combination of cisplatin and CQ dramatically increased LC3B-II in all treatment groups (Fig. 3D). Moreover, ATG13 protein expression slightly decreased in the cisplatin and CQ treated groups (Fig. 3D, Suppl. Figure 2D).

While the number of autophagosomes significantly increased following cisplatin treatment (Fig. 2B), the cisplatin + CQ combination led to a marked decrease in autophagosomes, suggesting that suppression of autophagy flux impaired the autophagic process (Fig. 3E, F).

In addition, we investigated the effects of autophagy inhibition on the stimulation of interferon regulated genes. Our qRT-PCR analysis revealed that IFIT2, IL-6 and ISG15 expression were significantly decreased in all Cis. + CQ combine treatment groups (Fig. 3G). For IFI44 mRNA, comparison between IC_25_ vs IC_25_ + CQ groups was not significant; however significant decreases were observed for IC_50_ vs IC_50_ + CQ (p = 0.0072) and IC_75_ vs IC_75_ + CQ (p = 0.0046) comparisons (Fig. 3G). These findings demonstrate that inhibition of autophagy leads to suppression of STING expression and the interferon response following cisplatin treatment.

### Impact of induction of autophagy

**Fig. 4:**
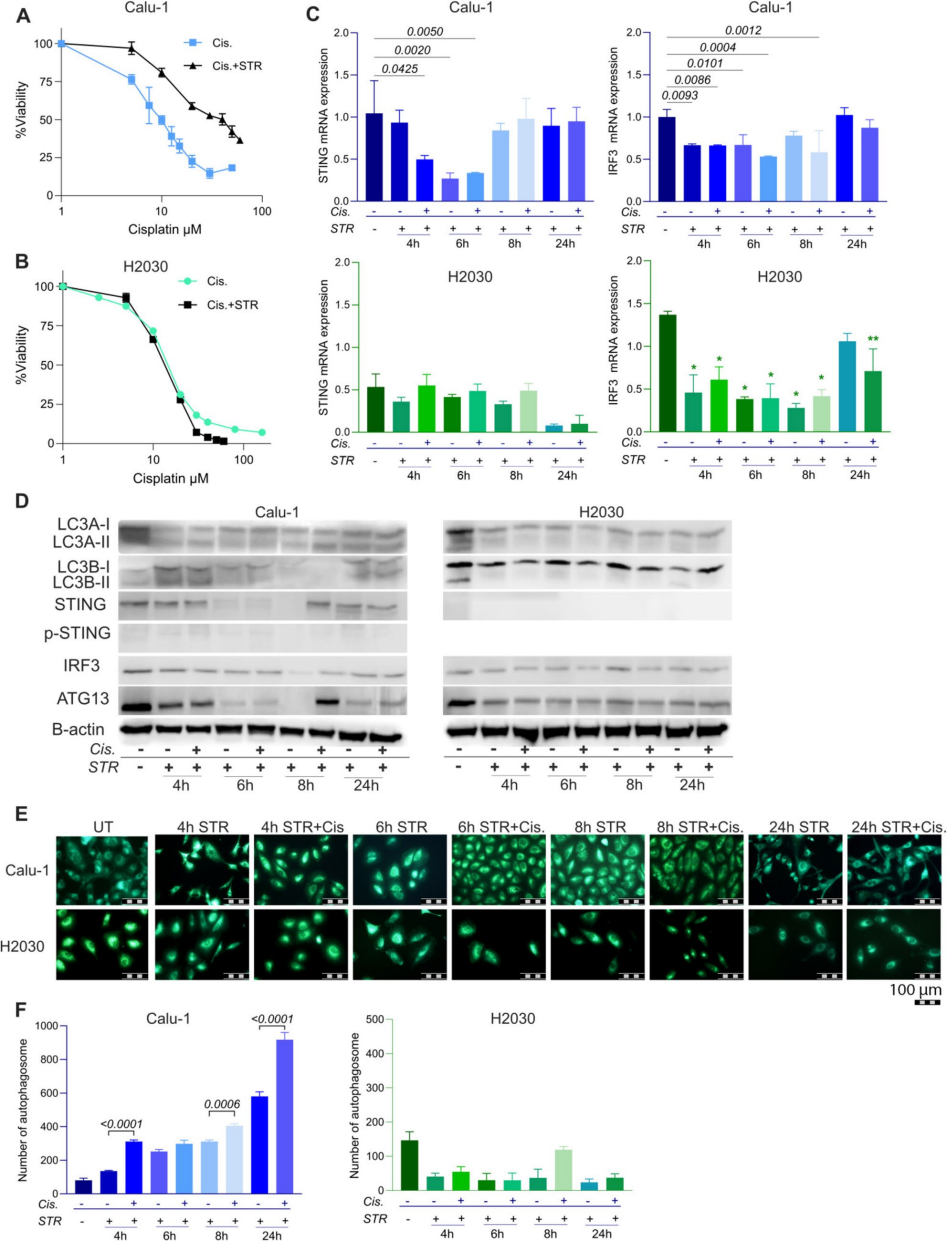
Impact of induction of autophagy on cisplatin sensitivity and STING expression. Cytotoxic effects of cisplatin in serum-starved **A** Calu-1, and **B** H2030 cells. Cisplatin treatments were applied at concentrations of 60-50-40-30-20-10-5 μM for 72 h.; **C** qRT-PCR results of STING and IRF3 gene expressions. p values for significant comparisons are indicated on the graphs using ANOVA and Dunnett’s multiple comparisons test. (*:p < 0.0001, **:p = 0.0002 for IRF3). The IC_50_ concentration was used for cisplatin treatments; **D** Western blot result of LC3A, LC3B, STING, p-STING, IRF3, and ATG13 proteins in Calu-1 and H2030 cell lines; **E** MDC staining of serum-starved and cisplatin treated cells, Scale bar = 100 µm.; **F** Bar graphs showing the number of autophagosomes

To examine the effects of stimulated autophagy on cisplatin sensitivity, STING expression, and interferon response, starvation (STR) experiments were performed. In Calu-1 cells, IC_50_ concentration of cisplatin increased from 9.16 µM to 36.35 μM after starvation (Fig. 4A). In contrast, starvation did not affect IC_50_ value of cisplatin in H2030 cells (Fig. 4B).

In Calu-1 cells, compared to untreated controls, cisplatin and starvation both reduced STING and IRF3 mRNA levels after 4 and 6 h of treatment, with expression returning to basal levels by the 8th hour (Fig. 4C). Although MDC staining indicated the presence of autophagosomes after starvation, western blot analysis did not show a significant increase in LC3B-II levels, suggesting limited autophagic flux. Consequently, no significant differences were observed when comparing the STR and Cis. + STR groups. Western blot analysis confirmed STING depletion at 6 h and a decrease in IRF3 protein expression at 8 h in Calu-1 cells (Fig. 4D, Suppl Fig. 3). A slight p-STING expression was observed at 4 and 6 h (Fig. 4D, Suppl Fig. 3).

For H2030 cells, starvation did not induce STING protein expression (Fig. 4D), therefore we did not analyse p-STING in H2030 cells. STING mRNA was detected at very low levels with no differences between the groups (Fig. 4C). Although IRF3 mRNA expression in H2030 cells decreased with starvation and cisplatin compared to control, no significant differences were observed between the groups. Similarly, IRF3 protein expression in H2030 cells exhibited similar patterns across all groups (Fig. 4D, Suppl. Figure 4).

Western blot analysis showed that LC3B-I and LC3B-II levels increased after starvation; however, cisplatin exposure did not affect the expression of LC3B in Calu-1 cells. Over time, LC3B accumulation did not continue (Fig. 4D, Suppl. Figure 3). LC3A-I and LC3A-II expressions were similar between cisplatin-treated and untreated starvation groups for both Calu-1 and H2030 cells (Fig. 4D, Suppl Figs. 3, 4). On the other hand, starvation and cisplatin exposure did not lead to LC3B-II accumulation in H2030 cells. ATG13 protein was re-expressed after being depleted over time in Calu-1 cells; whereas in H2030 cells, ATG13 levels slightly decreased compared to the control group and remained at a stable level (Fig. 4D, Suppl. Figures 3, 4).

MDC staining of autophagosomes revealed that starvation increased the number autophagosomes and cisplatin treatment further increases their number in Calu-1 cells (Fig. 4E, F). In contrast, starvation decreased the number autophagosomes in H2030 cells.

**Fig. 5:**
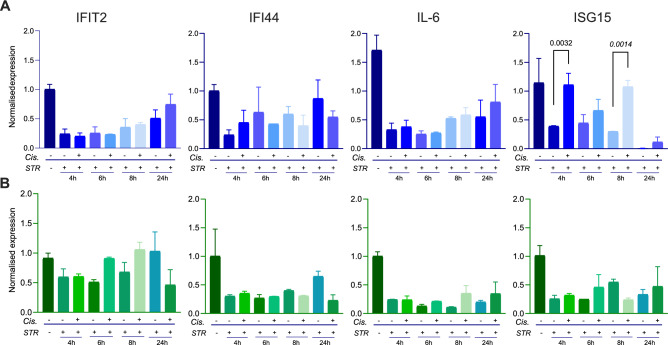
Impact of induction of autophagy on interferon response. qRT-PCR results of IFIT2, IFI44, IL-6 and ISG15 gene expressions in serum-starved and cisplatin treated **A** Calu-1, and **B** H2030 cells. The IC_50_ concentration was used for cisplatin treatments. p values for significant comparisons were calculated using ANOVA and Dunnett’s multiple comparisons test

Regarding interferon-stimulated gene (ISG) expression, we observed that cisplatin treatment increased the starvation-inhibited ISG15 expression in Calu-1 cells (Fig. 5A). The expression levels of IFIT2, IFI44, and IL-6 were similar between cisplatin-treated and untreated serum-starved Calu-1 and H2030 cells (Fig. 5).

## Discussion

In this study we report that cisplatin exposure increases STING expression, activates the cGAS-STING pathway and induces an interferon response in epidermoid carcinoma NSCLC cells. Additionally, our findings indicate that cisplatin induces autophagy in these cells, and when autophagy is pharmacologically inhibited, STING expression and interferon response decreases. Conversely, the adenocarcinoma cell line exhibited different patterns regarding STING expression, autophagy and interferon response. Specifically, cisplatin did not increase STING expression or interferon response in these cells, nor did it affect autophagy. We hypothesize that this difference may be due to the significantly higher STING expression in Calu-1 cells compared to H2030 cells.

Previous studies have shown that STING directly interacts with LC3 through its LC3-interacting region (LIR), thereby inducing autophagy [22]. Inhibition of autophagy reduces STING-LC3 interaction, thereby diminishing autophagy. These findings align with the primitive roles of the cGAS-STING pathway in innate immunity and evolutionarily conserved defence mechanisms [23]. Gui et al. reported that initiating autophagy is an ancient and conserved function of the cGAS-STING, and STING traffics to lysosomes via autophagosomes and endosomes [5]. Additionally, autophagy induced by STING activation generates negative feedback for the regulation of the immune response, thereby preventing excessive inflammatory damage [22]. These studies highlight the importance of autophagy-STING interactions in innate immunity which is essential for the body’s defence mechanisms.

Autophagy is a key regulator of cisplatin response in human cancers [24]. Consistent with previous studies, we observed that cisplatin exposure induces autophagy in Calu-1 cells [25]. However, H2030 cells did not exhibit this effect. Although autophagy has been reported in H2030 cells in response to different drugs or stimuli [26, 27], its induction by cisplatin has not been previously documented. As STING has been reported to form membranes necessary for LC3 lipidation [5] the reduced autophagic response in H2030 cells may be related to very low STING mRNA and protein. However, further studies, such as rescue experiments, are needed to confirm this hypothesis.

In the context of cancer progression and chemotherapy response, both protective and detrimental effects of autophagy and the cGAS-STING pathway have been observed. Our results showed that following autophagy inhibition, Calu-1 cells became approximately 2 times sensitive to cisplatin. Supporting our findings, a study on ovarian cancer demonstrated that autophagy inhibition enhances cellular sensitivity to cisplatin [28]. Conversely, induction of autophagy resulted in approximately a 3.97-fold increase in resistance to cisplatin in Calu-1 cells. Similar results have been reported in ovarian cancer cells, where increased autophagy was associated with the development of cisplatin resistance [29]. Furthermore, it has been reported that cisplatin-induced DNA damage increases STING expression and activates the cGAS-STING pathway [15]. Our study confirms that cisplatin induces STING transcription in Calu-1 cells in a dose- and time-dependent manner. Moreover, inhibition of autophagy reversed cisplatin-induced STING upregulation. In line with our findings, Bai et al. reported that anticancer drugs induce mitotic catastrophe thereby activating the cGAS-STING pathway. Additionally, they demonstrated that autophagy inhibition enhances mitotic catastrophe-induced anticancer effects by modulating the cGAS-STING pathway [30].

In cancer cells, autophagy can function as a drug resistance mechanism, allowing cells to evade apoptosis. Consequently, the inhibition of autophagy in the presence of oncogenic mutations such as p53 and KRAS has been investigated to develop personalized treatments. However, KRAS mutations have not been found to significantly impact the sensitivity of cancer cells to autophagy inhibition [31] and cisplatin-induced autophagy appears similar between p53 wild-type and knockout cells [32]. In this study, we used Calu-1 cell line which lack p53 expression due to a homozygous deletion and H2030 cells which harbour a KRAS mutation. We found that cisplatin treatment induces STING expression, interferon response, and autophagy in Calu-1 cells. In contrast, these effects were absent in H2030 cells, even though autophagy induction by starvation in H2030 cells resulted in only a limited increase in autophagosome formation. Therefore, we suggest that differences in STING protein expression may account for the observed variations in autophagy patterns between these two cell lines.

A limitation of our study is that we did not determine the IC_50_ value of chloroquine (CQ) alone under our experimental conditions. Although CQ was used at a concentration (25–50 µM) widely reported to have minimal cytotoxic effects in NSCLC cell lines [33], future studies assessing CQ cytotoxicity directly would strengthen the interpretation of the observed combinatorial effects with cisplatin. Another limitation of this study is the absence of genetic modulation experiments (e.g., STING knockdown via siRNA or shRNA). As this project was part of a master’s thesis with limited experimental scope, further studies are currently planned to perform targeted genetic interventions to better define the role of STING in regulating autophagy and cisplatin sensitivity in NSCLC cells.

Collectively, our findings highlight the critical role of autophagy in cisplatin’s mechanism of action and suggest that STING protein expression and cGAS-STING pathway activation enhance cisplatin’s chemotherapeutic efficacy. However, our study has several limitations. Further research involving STING knockout and wild-type cells, as well as studies examining the effects of different autophagy inducers and inhibitors, will be necessary to fully elucidate these mechanisms. Such investigations may contribute to the development of more effective cisplatin-based cancer treatments.

## Supplementary Information

Below is the link to the electronic supplementary material.

Supplementary file1 (DOCX 1143 KB)

## Data Availability

No datasets were generated or analysed during the current study.
