# Supplementary material for: Differential regulation of STING expression and cisplatin sensitivity by autophagy in non-small cell lung cancer cells
Source: Med Oncol. 2025 May 30;42(7):227. doi: 10.1007/s12032-025-02786-2 (PMC12125072; doi:10.1007/s12032-025-02786-2)
Supplement: Supplementary file 1 — Supplementary file1 (DOCX 1143 KB) [file 12032_2025_2786_MOESM1_ESM.docx]

**Supplemental Figures**

**Differential Regulation of STING Expression and Cisplatin Sensitivity by Autophagy in Non-Small Cell Lung Cancer Cells**

Sevim Aydemir, Zafer Yildirim, Busra Bara, Eda Dogan, Vildan Bozok*

Ege University, Faculty of Medicine, Department of Medical Biology, Izmir, Turkiye


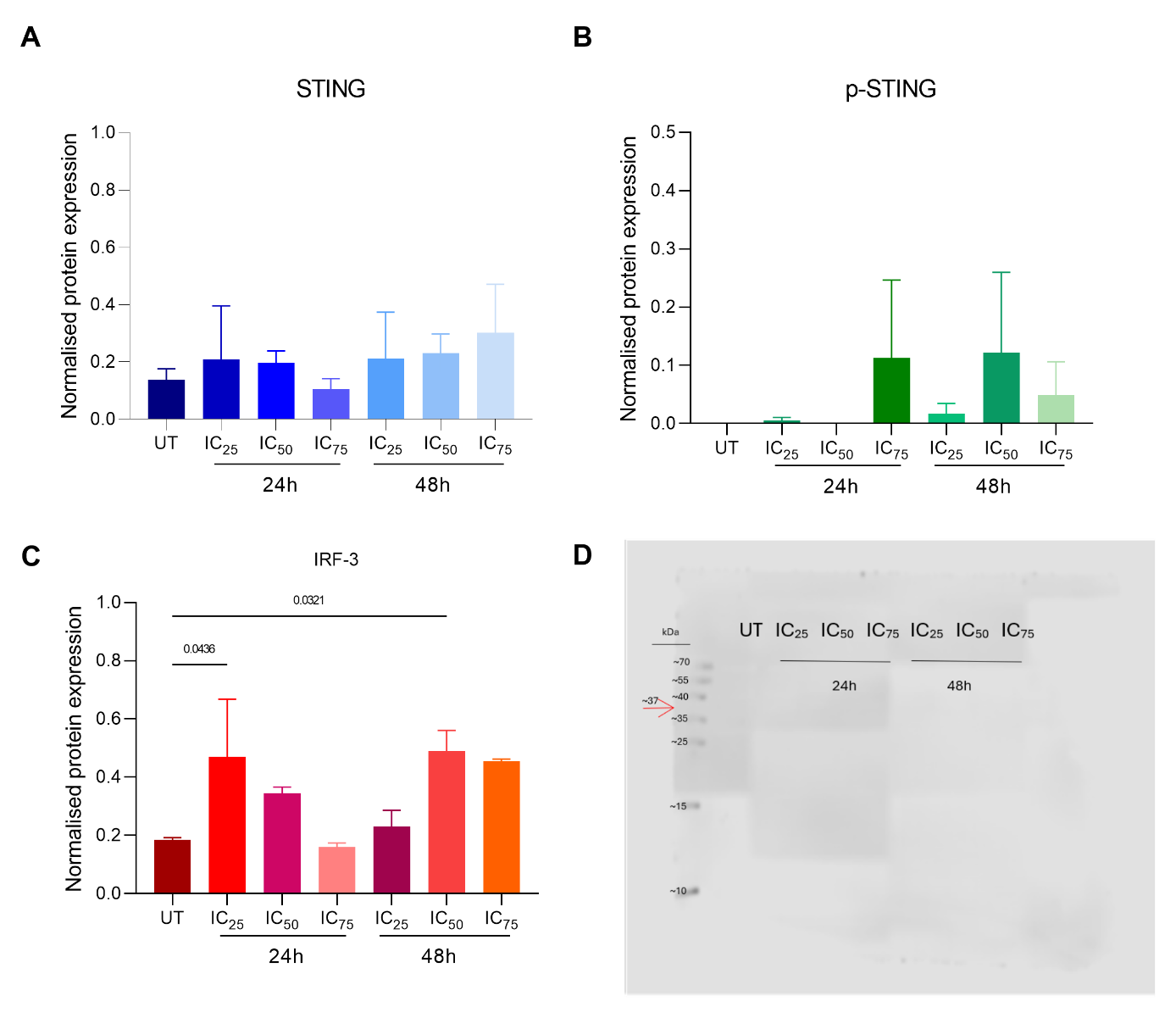


**Supplemental Figure 1**: **Cisplatin treatment induces STING expression in epidermoid carcinoma but not adenocarcinoma NSCLC cells.** Quantification of western blot results of Calu-1 cells using ImageJ; **A)** STING, **B)** p-STING, **C)** IRF-3 proteins. **D)** Western blot membrane of STING protein for H2030 cells. IC_25_, IC_50_ and IC_75_ treatment doses are 4.58 µM, 9.16 µM and 13.75 µM for Calu-1 and 6.76 µM, 13.52 µM and 20.28 µM for H2030 cells, respectively.


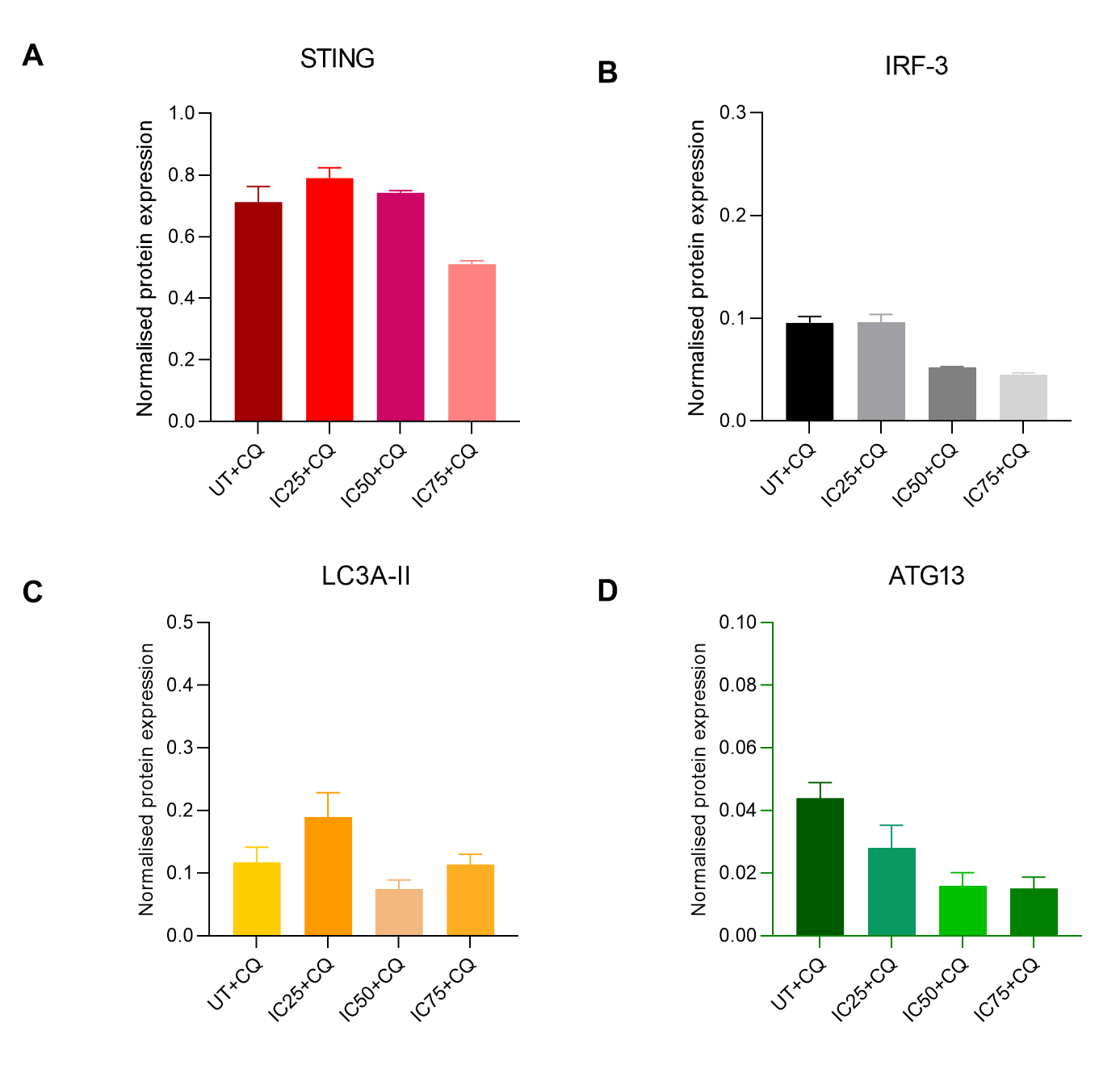


**Supplemental Figure 2**: **Impact of inhibition of autophagy on cisplatin sensitivity, STING expression and interferon response.** Quantification of western blot results of Calu-1 cells using ImageJ. **A)** STING **B)** IRF-3, **C)** LC3A-II and **D)** ATG13. Cisplatin concentrations: 4.58, 9.16, 13.75 µM for IC_25_, IC_50_ and IC_75_ groups, respectively. CQ dose: 50 μM.


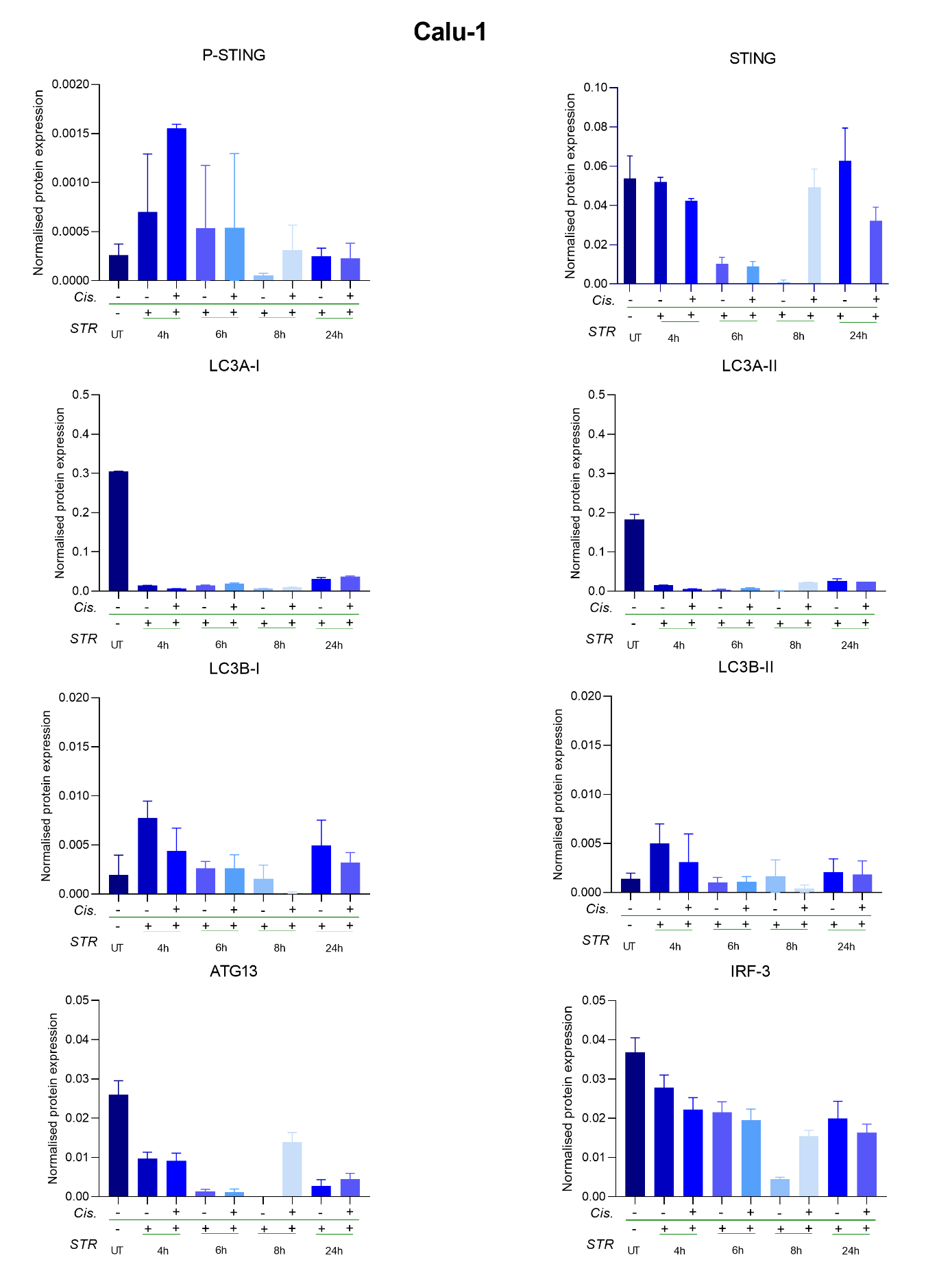


**Supplemental Figure 3: Impact of induction of autophagy on cisplatin sensitivity.** Quantification of western blot results using ImageJ in Calu-1 cell line.


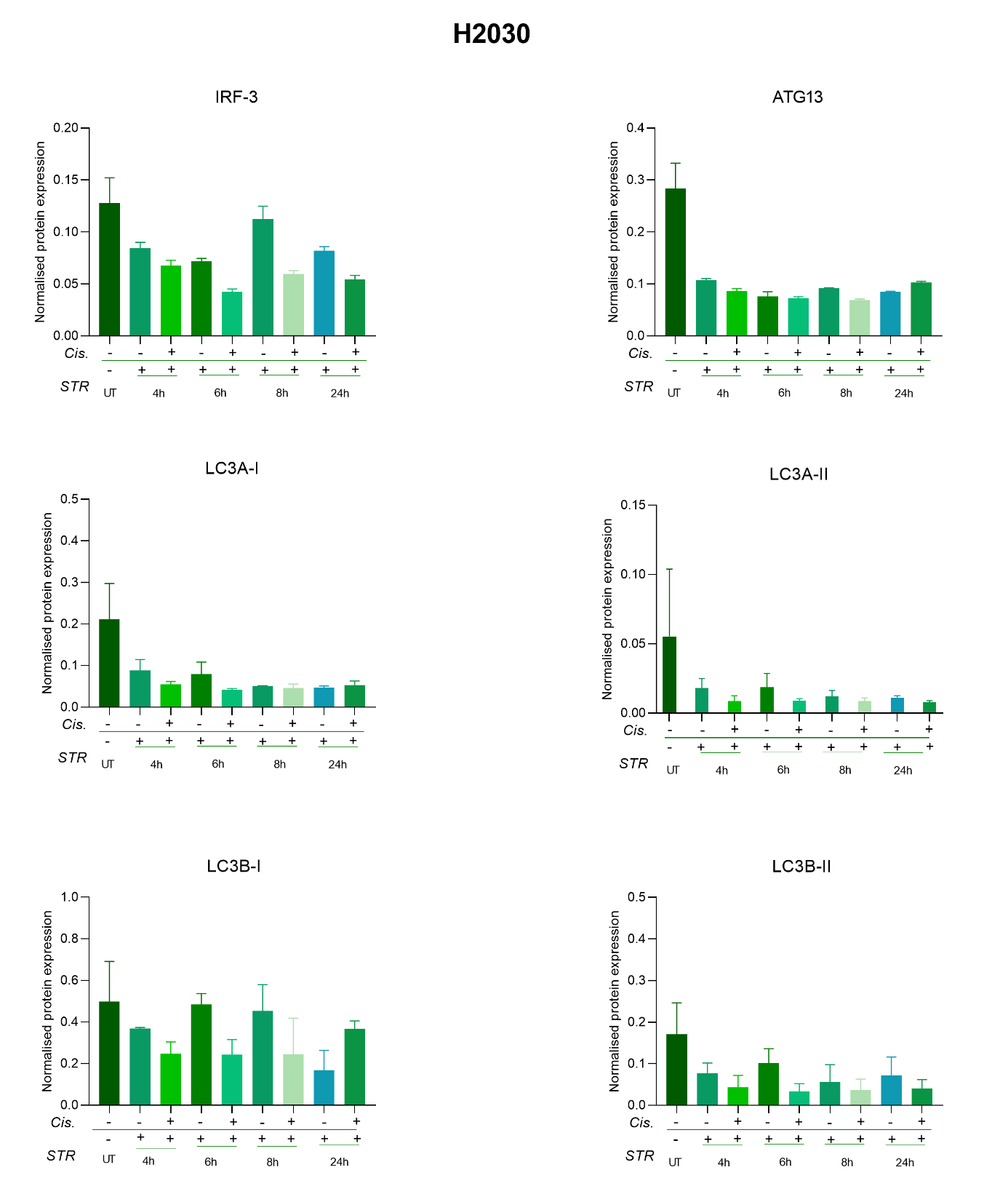


**Supplemental Figure 4: Impact of induction of autophagy on cisplatin sensitivity.** Quantification of western blot results using ImageJ in H2030 cell line.
